# Postoperative pain relief using intermittent intrapleural analgesia following thoracoscopic anterior correction for progressive adolescent idiopathic scoliosis

**DOI:** 10.1186/1748-7161-8-18

**Published:** 2013-11-16

**Authors:** Stephen AC Morris, Maree T Izatt, Clayton J Adam, Robert D Labrom, Geoffrey N Askin

**Affiliations:** 1 QUT/Mater Paediatric Spine Research Group, Queensland University of Technology and Mater Research, Level 2, Aubigny Place, Raymond Terrace, South Brisbane, Queensland 4101, Australia; 2 Institute of Health and Biomedical Innovation, Queensland University of Technology, Brisbane, Queensland, Australia

**Keywords:** Adolescent idiopathic scoliosis, Thoracoscopic anterior spinal fusion, Anterior fusion, Intrapleural analgesia, Endoscopic anterior surgery, Pain relief, Scoliosis surgery

## Abstract

**Background:**

Thoracoscopic anterior scoliosis instrumentation is a safe and viable surgical option for corrective fusion of progressive adolescent idiopathic scoliosis (AIS) and has been performed at our centre on 205 patients since 2000. However, there is a paucity of literature reporting on or examining optimum methods of analgesia following this type of surgery. A retrospective study was designed to present the authors’ technique for delivering intermittent local anaesthetic boluses via an intrapleural catheter following thoracoscopic scoliosis surgery; report the pain levels that may be expected and any adverse effects associated with the use of intrapleural analgesia, as part of a combined postoperative analgesia regime.

**Methods:**

Records for 32 patients who underwent thoracoscopic anterior correction for AIS were reviewed. All patients received an intrapleural catheter inserted during surgery, in addition to patient-controlled opiate analgesia and oral analgesia. After surgery, patients received a bolus of 0.25% bupivacaine every four hours via the intrapleural catheter. Patient’s perceptions of their pain control was measured using the visual analogue pain scale scores which were recorded before and after local anaesthetic administration and the quantity and time of day that any other analgesia was taken, were also recorded.

**Results:**

28 female and four male patients (mean age 14.5 ± 1.5 years) had a total of 230 boluses of local anaesthetic administered in the 96 hour period following surgery. Pain scores significantly decreased following the administration of a bolus (p < 0.0001), with the mean pain score decreasing from 3.66 to 1.83. The quantity of opiates via patient-controlled analgesia after surgery decreased steadily between successive 24 hours intervals after an initial increase in the second 24 hour period when patients were mobilised. One intrapleural catheter required early removal due to leakage; there were no other associated complications with the intermittent intrapleural analgesia method.

**Conclusions:**

Local anaesthetic administration via an intrapleural catheter is a safe and effective method of analgesia following thoracoscopic anterior scoliosis correction. Post-operative pain following anterior thoracic scoliosis surgery can be reduced to ‘mild’ levels by combined analgesia regimes.

## Background

**Figure 1:**
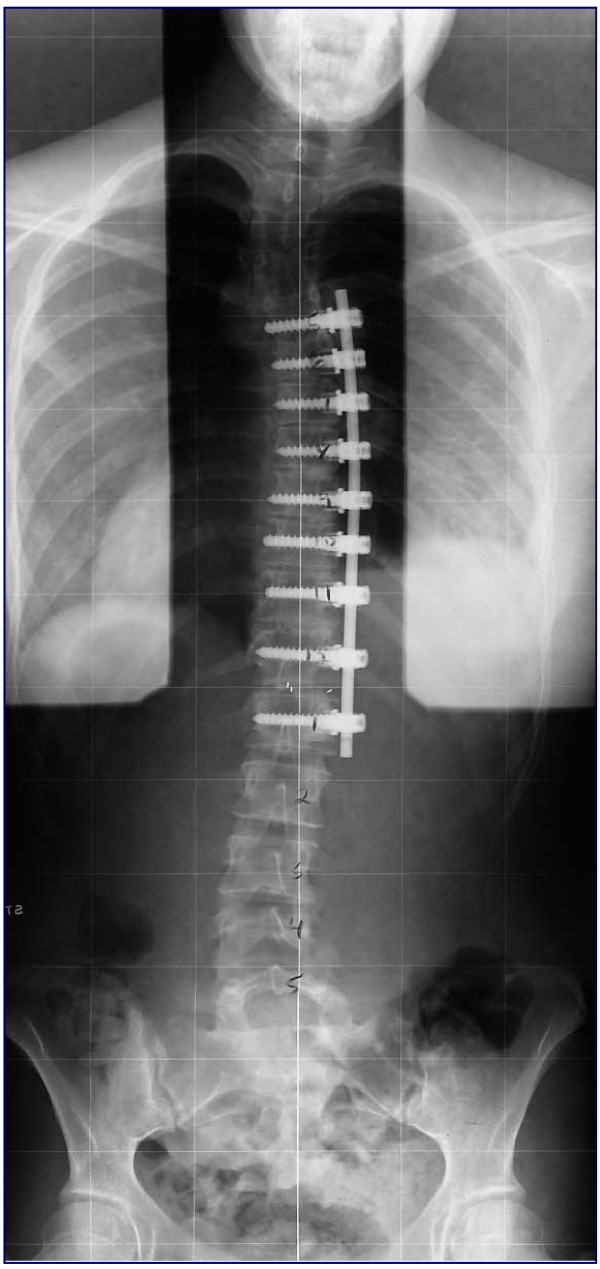
Post-operative plain standing radiograph following thoracoscopic anterior spinal fusion for progressive adolescent idiopathic scoliosis.

Thoracoscopic anterior scoliosis correction is a safe and viable surgical option for corrective fusion of progressive adolescent idiopathic scoliosis [1-3], see Figure 1. Reported benefits include; improved cosmesis, sagittal profile restoration, faster recovery of pulmonary and shoulder girdle function, and a less invasive surgical approach when compared to open thoracotomy or posterior spinal fusion [4-7]. This has the potential to reduce the intensity and duration of post-operative pain, in addition to the quicker recovery of function stated previously. However, there is a paucity of research in the literature reporting on or examining analgesia methods and pain relief outcomes following thoracoscopic anterior scoliosis fusion (TASF).

The role of analgesia following thoracoscopy and thoracotomy for pulmonary pathology has formed an important part of the cardiothoracic literature. Intrapleural and paravertebral analgesia have been successfully used following cardiothoracic procedures involving thoracotomy [8-11], thoracoscopy [12-15] and video-assisted thoracic surgery [16]. These techniques have demonstrated good efficacy when compared to epidural use [17], and a review by De Cosmo *et al*. [18] concluded that whilst epidural is the gold standard, paravertebral block is an effective alternative for post thoracotomy analgesia and maintains pulmonary function better. It is however, not possible to directly relate the results from cardiothoracic studies to spinal deformity surgery since those procedures often do not involve division of the postero-medial parietal pleura, performing discectomies, or instrumenting the spine. Another consideration, following anterior thoracic scoliosis correction, is differentiating between an intrapleural and a paravertebral effect; the paravertebral space lies posterior to the parietal pleura and anterior to the superior costo-transverse ligament, whilst the intrapleural space lies between the visceral and parietal pleura. Since the parietal pleura is disrupted during thoracic scoliosis correction, local anaesthetic may move between these two spaces creating a dual effect.

Following spinal deformity correction, most previous literature has looked at analgesia methods after posterior instrumented fusion, particularly using patient-controlled analgesia, epidural, or local anaesthetic infiltration [19,20]. Following anterior scoliosis correction, there has been very limited work looking at any type of analgesia methods; epidural use has been found to be safe when inserted under direct vision after rib head resection via an open thoracotomy [21] and to give better post-operative analgesia with fewer side-effects, when compared to intravenous morphine [22]. The thoracic epidural catheter can be placed pre-operatively [23] but it is challenging with potential risks due to the scoliotic bony deformities and at times may be impossible due to the concomitant flattening or reversal of the normal sagittal curves in scoliosis patients. This difficulty resulted in the intrapleural analgesia technique being adapted for use at our centre and used thereafter for all TASF procedures over ten years ago. To our knowledge, the use of intrapleural or paravertebral blocks following open anterior or thoracoscopic anterior scoliosis correction has not been described to date.

Accordingly, the aims of this retrospective case series analysis were to present the authors’ technique for delivering intermittent local anaesthetic boluses via an intrapleural catheter following thoracoscopic scoliosis surgery; report the pain levels that may be expected and any adverse effects associated with the use of intrapleural analgesia following TASF as part of a combined postoperative analgesia regime.

## Methods

A subset of the most recent 80 patients from a large, single centre consecutive series of 205 patients who had undergone TASF for progressive adolescent idiopathic scoliosis had their medical records reviewed. As the study involved the use of an existing database and the data being retrospectively collected from medical records was non-identifiable data, it was considered a clinical audit and exempt from requiring ethical review. The surgeries were all performed by the two senior authors (GNA and RDL) at the Mater Children’s Hospital, Brisbane, Australia. The indication for this type of surgery as opposed to a posterior selective thoracic fusion were: Lenke type 1 with thoracic Cobb angle between 40 - 70°, minimum of 50% correction of main thoracic curve on fulcrum bending radiograph, correctibility of any lumbar compensatory curve on side bending radiograph, and T5-T12 thoracic kyphosis angle of < 40°. Patient and deformity correction details were prospectively recorded in the departmental surgical database for all cases, from the larger patient series. The surgical procedure was based on the technique first described in 2001 by Picetti *et al.*[24], and subsequently by Norton *et al.*[1] and Gatehouse *et al.*[25]. After deflation of a single lung, four portals were made, with the initial most proximal portal being used to assist additional portal placement under direct visualisation using the endoscope. The portals are positioned based on the desired trajectory of the screws which is to be parallel to the vertebral endplates. The portals were made between the ribs through a split made in the intercostal muscles, usually two intercostal spaces apart, allowing access to two or three vertebral levels from a single portal. No ribs are resected or removed during portal placement. If the instrumentation was to extend beyond T12, an interbody spacer cage packed with graft material was placed between T12-L1 after discectomy to assist the spine’s transition into lordosis. Generally L1 can be reached consistently from the thoracic cavity. If the diaphragm inserts above the T12-L1 disc, the diaphragm was partially dissected to allow access from the thoracic cavity. After visualization of lung reinflation, a 20 French Gauge chest drain was inserted through a small additional anterior incision (used for the suction apparatus during the procedure), before closure of the final distal operative portal. Following surgery, all patients received local anaesthetic analgesia via an intrapleural catheter (16 Gauge Portex Epidural Catheter, Smiths Medical Australasia Pty. Ltd, Brisbane, Australia), as well as intravenous opiate patient-controlled analgesia (PCA) and non-opiate oral analgesia.

**Figure 2:**
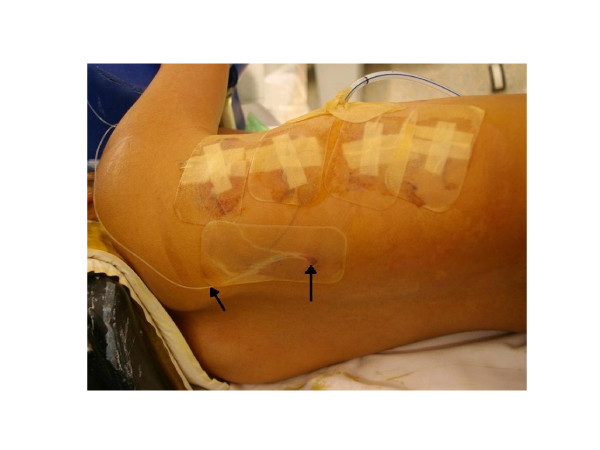
Intra-operative photograph demonstrating the intrapleural catheter (marked by two arrows) exiting through the right paraspinal region, posterior to the thoracoscopy portals and intercostal chest catheter.

**Figure 3:**
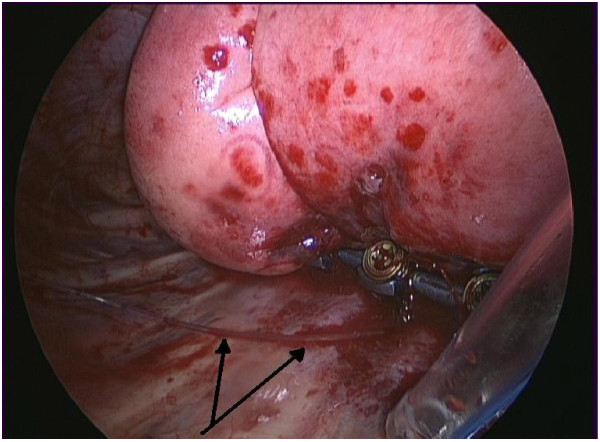
Intra-operative photograph showing the intrapleural catheter (marked with two arrows) lying on the posterior chest wall, adjacent to the spinal instrumentation and intercostal catheter.

The technique for using intrapleural analgesia was as follows: an intercostal epidural catheter was percutaneously inserted under direct vision at the end of the surgical fusion (Figure 2), using a needle introducer prior to lung re-inflation (Figure 3). Efforts are made to position the intrapleural catheter behind the parietal pleura with the tip delivered at least 5cm into the sub-parietal potential space towards the superior end of the spinal instrumentation. The bupivacaine bolus is therefore somewhat contained, to allow the anaesthetic to infiltrate and soak the region where the intercostal nerves exit the spine. With the pleural effusion pooling inferiorly in the chest, it is expected there would be a minimal dilutional effect on the local anaesthetic bolus. A bolus of Bupivacaine (1mg/kg in 20-25mls of 0.9% Saline) was then given approximately every four hours until it was removed. To avoid the local anaesthetic being taken immediately by the chest drain, the chest drain was clamped prior to the injection of the bolus and remained clamped for a subsequent 20 minutes, under close nursing observation. The relevant section of the policy document provided to nursing staff pertaining to the delivery of the intrapleural bolus and monitoring of the patient is provided as Table 1. Regular Paracetamol and Ibuprofen or Diclofenac were also given. Additionally, an intravenous opiate PCA was set up immediately post-operatively and was available to the patient for breakthrough pain, as required, until the patients’ analgesia requirements were at a level where the PCA could be removed.

**Table 1:** Selected section of “intermittent bolus of intrapleural analgesia policy document pertaining to delivery of bolus dosing regime and monitoring of the patient” document provided to nursing staff

**Delivery of bolus dosing regime**	
1.	Do baseline blood pressure, heart rate, respiratory rate, oxygen saturations, sedation score and pain assessment
2.	Position patient operated side up prior to administration of the bolus dose
3.	Clamp the thoracic pigtail drain and/or intercostal catheter if in situ
4.	Independently double check the pre-programmed bolus against the prescription and patient with a 2nd Registered Nurse
5.	Administer the bolus dose (The time of dose delivery is volume dependant and may take as long as 12 minutes to deliver)
6.	Continue observations as above every 5 minutes for the next 30 minutes. But after 20 minutes from commencement of bolus delivery, unclamp the pigtail drain and/or intercostal catheter
7.	Document and sign the Pain Management Prescription form
8.	Observe the drain to ensure patency
9.	Position patient in a comfortable position
10.	Perform further observations as above after 30 minutes.
11.	Then hourly observations until the next bolus is due (excluding the Blood Pressure)
**Monitoring of the patient**	
1.	Observations are to be performed as detailed above for delivery of a bolus
2.	More frequent observations are required if clinically indicated or as stated in each child’s care plan. Observations include:
	· Respiratory rate
	· Heart rate
	· Blood pressure - not required 1 hour after bolus delivery unless clinically indicated
	· Pain score at rest and on movement (not required if the patient is sleeping)
	· Continuous oxygen saturations
	· Sedation score
	· Cumulative dose/deliveries
	· Any adverse events
	· Temperature and site check every 4 hours
**Adverse events**	
1.	If adverse events occur during a bolus, cease the bolus immediately.
	Notify Acute Pain Service (APS)/Anaesthetist for all Adverse Events:
	· Horner’s Syndrome – facial palsy, droopy eyelid, red eye, hoarse voice
	· Hypotension
	· Pneumothorax
	· Infection
	· Pain score greater than or equal to 4/10, check catheter, give multi-modal analgesia
	· Leaking at the wound - reinforce if sterility maintained
	· Disconnected catheter - cease infusion, wrap in sterile gauze keeping the catheter tip below the level of the incision, contact APS and inform Surgical Team of the APS decision )
	· Signs and symptoms of local anaesthetic toxicity are:
	➢ Facial tingling/numbness
	➢ Tinnitus
	➢ Metallic Taste
	➢ Twitching
	➢ Seizures
	➢ Apnoea
	➢ Hypotension
	➢ Cardiac arrhythmia/arrest
2.	In the event of an emergency (including signs and symptoms of local anaesthetic toxicity)
	· Stop the infusion immediately/unclamp chest drain if applicable
	· Press emergency button, call emergency number
	· Give oxygen/resuscitate if necessary
	· Obtain intralipid 20% from Paediatric Intensive Care Unit
	· Notify APS/Anaesthetist

In the immediate post-operative period, all patients provided self-assessment of their pain levels using a visual analogue pain score from zero to ten. This was recorded by nursing staff in the patients’ medical records every hour when awake. For inclusion in the study, patients were required to have pain scores recorded within one hour prior to local anaesthetic intrapleural bolus and between one and two hours following the bolus. These inclusion criteria were used to allow statistical analysis of changes in pain score before and after the intrapleural bolus. Details (date, time and dose) regarding any use of opiates via patient-controlled intravenous administration and other oral analgesics were also recorded. Any adverse events related to analgesia were identified by reviewing medical and nursing entries in the patients’ medical records.

Data analysis was undertaken using SPSS Version 17 (Chicago, IL, USA). Pre- and post-bolus pain scores were compared using paired t-tests. Day to day changes in opiate usage were also assessed using paired t-tests, as was the mean hourly opiate usage before and after intrapleural catheter removal.

## Results

Of the 80 medical records retrieved, 32 patients had sufficient data for inclusion in the study. There were 28 females and four males with a mean age of 14.5 ± 1.5 years (range, 11.6-18.0). For the study cohort, a total of 230 boluses of local anaesthetic were administered via an intrapleural catheter for a period of 58.3 ± 16.4 hours (range, 26-96) after surgery prior to its removal. One intrapleural catheter required early removal at 26 hours after surgery, due to leakage. The chest drain was removed at mean 61.1 ± 13.1 (median 59, range 48 – 91) hours after surgery when the haemoserous exudate was draining at less than 150 mls per day.

The remaining 48 patients (45 females, three males) reviewed, where data was insufficient for inclusion in the analysis, were similar to the study group of 32, with mean age 16.0 ± 1.8 years (range, 13.3-20.4). For this group, the chest drain was removed similarly at mean 60.0 ± 15.7 (median 48, range 48 – 96) hours after surgery. For this group it was noted there were no adverse events or complications relating to their intrapleural analgesia.

**Figure 4:**
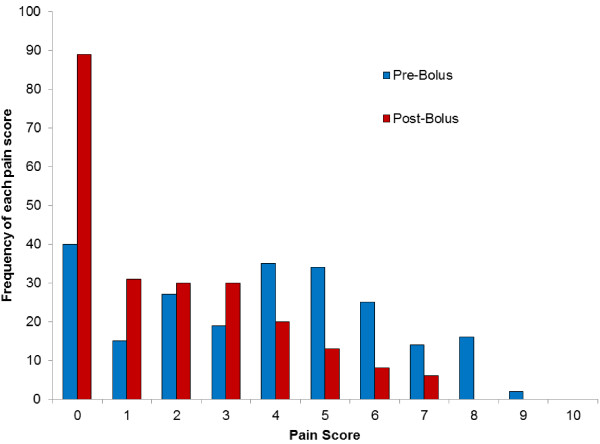
Histogram demonstrating the frequency that each possible pain score was reported throughout the hospital stay, before and after administration of the local anaesthetic bolus following thoracoscopic anterior spinal fusion surgery (total 230 boluses).

**Figure 5:**
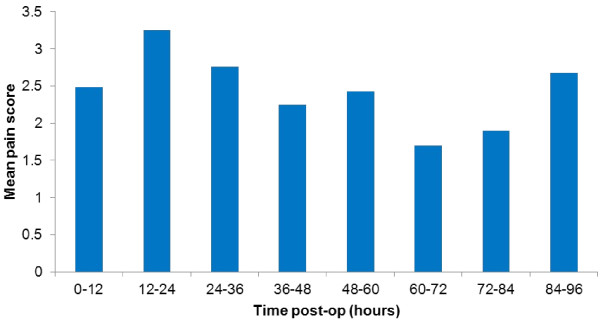
Mean pain scores (scale 1-10) for all patients during the first four postoperative days after thoracoscopic anterior spinal fusion surgery.

Pain scores significantly decreased following administration of an intrapleural bolus (p < 0.0001) with the overall mean pain score decreasing from 3.66 to 1.83. Figure 4 gives the frequency distribution of each pain score before and after administration of a local anaesthetic bolus, clearly showing the shift toward lower pain scores which occurs post-bolus. The mean pain scores for all patients during the first four post-operative days are shown in Figure 5.

**Figure 6:**
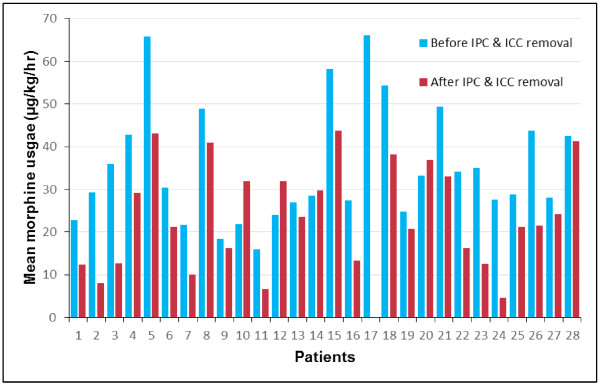
**Mean hourly opiate usage before and after removal of intrapleural catheter (IPC) and intercostal catheter (ICC).** Note patient 17 had their PCA removed at the same time as their IPC and ICC. 4 patients were excluded from this analysis since their PCA had been changed to fentanyl due to nausea from morphine.

The PCA was removed at mean 79.4 ± 16.5 (median 73.0, range 41.0 – 118.0) hours after surgery. It remained in use for a longer duration than the intrapleural catheter in 27 patients; of the remainder, two patients had their PCA removed first, whilst three patients had the PCA removed at the same time as the intrapleural catheter. The mean quantity of opiate PCA used within each successive 24 hour interval during the post-operative period is shown in Table 2 with each period found to be statistically significantly different to the time period prior. Mean hourly intravenous opiate use was found to be lower again once the intrapleural catheter had been removed, with a reduction in mean usage from 35.3 to 23.1 μg/kg/hour (p = 0.0001). Figure 6 presents the mean hourly opiate usage before and after removal of the intrapleural catheter for all patients. There were no complications or adverse events relating to analgesia delivery methods found for the study group or in any of the total 80 reviewed medical records. On questioning the surgeons regarding the entire cohort (205 cases) since the intrapleural analgesia technique was introduced, neither surgeon could recall a failure or adverse event related to the method which in part contributed to their desire to publish on our experience with the technique.

**Table 2:** Changes in the quantity of opiate patient-controlled analgesia (PCA) usage between successive 24 hours intervals, during the first 96 hours following TASF (SD, standard deviation)

**After surgery**	**Mean opiate usage**	**Difference**	**Significance**
**(Hours)**	**(μg/kg) ± SD**		**(p < 0.01)**
0 – 24	445 ± 365		
24 – 48	841 ± 300	Increased	p < 0.0001
24 – 48	841 ± 300		
48 – 72	565 ± 307	Decreased	p = 0.0002
48 – 72	565 ± 307		
72 – 96	238 ± 314	Decreased	p < 0.0001

## Discussion

Research examining analgesia efficacy following surgery is essential, in order to ensure optimum post-operative care is given to patients. Due to the difficulty of insertion and placement of the thoracic epidural in patients with significant thoracic scoliotic deformities requiring instrumented fusion, the intrapleural analgesia technique was adapted at our centre to deliver local anaesthetic directly to the spine after anterior thoracic fusion surgery. This retrospective study was performed to present the authors’ technique of using intermittent analgesia via an intrapleural catheter, present patient reported pains scores after thoracoscopic scoliosis correction and identify any adverse events related to the technique.

The use of an intrapleural catheter placed under direct vision in the operating theatre has several advantages; it allows the delivery of local anaesthetic directly to the site of the spinal levels involved in the instrumented fusion surgery and allows pre-emptive analgesia to be administered in these predominantly adolescent patients. Pre-emptive analgesia has been shown to have more beneficial effects and results in reduced analgesia dosages being used, as opposed to waiting for pain levels to increase prior to giving analgesia [26-30]. Effective analgesia after open thoracic surgery is conducive to good respiratory effort, better ventilatory mechanisms and gas exchange and decreased incidence of atelectasis [18]. The respiratory benefits of effective analgesia are also highly desirable after minimally invasive surgery involving single lung ventilation, as well as for early mobilisation of these patients. A previous study on the first 100 cases of the larger patient series at our centre [25] found patients were mobilised a mean 1.6 days after surgery and was significantly less again when the initial 40 cases were excluded to account for the learning curve of the thoracoscopic technique and postoperative management regimes.

The technique of giving an intermittent bolus as opposed to a continuous infusion of local anaesthetic, allows the delivery of a higher dose of anaesthetic and therefore a more concentrated effect to achieve a dense neural block and is in keeping with postoperative epidural practice. The chest drain is clamped for around 30 minutes during delivery of the bolus and at all other times functions normally to assist with the clearance of the pleural effusion. A chest drain under continuous suction would hinder the effectiveness of a continuous delivery of local anaesthetic so was considered inappropriate for patients after TASF. Postoperatively, patients don’t tend to complain of pain from the incisions/portals over their general spine pain. However the intrapleural anaesthetic bolus is able to freely flow in the pleural space so may have some effect on minimising pain originating from the portals. With the patients receiving a multi-modal analgesia approach it is difficult to attribute how much the intrapleural analgesia may ease any portal incision pain over the effects of the PCA and additional non-opiate oral analgesia.

It is worth noting that for 42 of the 230 boluses (18%) assessed in the current study, the patients already had a pain score of zero before receiving a further local anaesthetic bolus, demonstrating good on-going analgesia between boluses. It is also important to note that, at our hospital, the use of intrapleural catheters and their analgesic effectiveness is carefully monitored on the ward by nursing, pharmacy and medical staff with the support of a specialised pain team. A protocol document to describe how to use the catheter, any potential complications, and the action to take in the event of any complication was developed (Table 1). Registered nurses caring for a patient with intermittent intrapleural analgesia must have attended a Paediatric Pain Management Workshop with successful completion of the two relevant modules (Management of a Paediatric Patient under the Pain Management Service, and Intermittent Bolus Intrapleural Analgesia).

The visual analogue pain score is a well-recognised tool for monitoring post-operative pain [31-33] and has been validated in the paediatric population [34]. It has previously been suggested that, on the 100 mm scale, a score of 0-4 mm can be considered pain-free, 5-44 mm mild pain, 45-74 moderate pain, and more than 75 mm is severe pain [35] Bird *et al.*[36] suggested that 13 mm was a significant decrease in scores less than 34 mm, whilst Bodian *et al.*[37] proposed that for pain scores between 3 and 7 on a ten-point scale, a change of one or more was significant. This supports the current study findings that the mean decrease in pain score (a decrease of 1.75 on the ten-point scale) after administration of an intrapleural bolus was indeed significant. However, this does not negate the fact that pain is a subjective sensation that has multiple influencing factors, along with the potential placebo effect from receiving a bolus of local anaesthetic. Figure 5 presents the mean pain scores for all patients during the first four postoperative days, with the generally low mean scores suggesting the analgesia regime was effectively controlling the pain of the patient group. With the chest drain removed at mean 61 hours after surgery, and the intrapleural catheter removed at mean 58 hours after surgery, these may both have contributed to the drop of mean pain scores in the 60-72 time period of Figure 5. The histogram in Figure 4 clearly shows the change in distribution of pain scores reported before and after intrapleural analgesia is given. Before a bolus, the pain scores given most often were at the higher end (4-6) of the VAS scale, with a shift of the distribution toward lower pain scores (0-3) after a bolus was given, with a substantial proportion of the group reporting zero pain scores after a bolus.

We note that the most common reason for pain scores not being recorded in the medical notes in this study was the patient sleeping. The protocol policy document instructs staff not to seek a pain score from a sleeping patient (Table 1). Missing scores are a potential problem for any prospective study, however waking a sleeping child for assessment of their pain level would predictably result in an inaccurate and anomalous score recorded. The fact that a patient was sleeping would suggest that the pain management was satisfactory and, therefore, conducive to the on-going sleep state.

Patients in the study received opiates via a patient-controlled intravenous method. The quantity of opiates used via PCA in this study increased significantly in the second 24 hour period after surgery. This period (24 – 48 hours postop) coincides with the patient being mobilised and sat out of bed for the first time since surgery and physiotherapy and nursing interventions commenced to achieve restoration of full lung re-expansion and volumes. Opiate usage decreased significantly between the second and third and third and fourth 24 hour periods and was lower again once the intrapleural catheter and chest drain were removed (Figure 6). This may suggest that the catheter and chest drains were a source of some pain but it is most likely a combination of the removal of these irritants and the normal course of rapid recovery after minimally invasive surgery. Patients often report some shoulder tip pain from the chest drain which does not respond to IV narcotics and is eased by the intrapleural bolus. The shoulder tip pain ceases when the chest drain is removed on Day 2 or Day 3. Patients are discharged home usually Day 5 after surgery and are routinely prescribed non-steroidal anti-inflammatories and paracetemol based medications. The steady reduction of opiate usage, shown in Table 2, was expected since the immediate post-operative pain would likely be improving with each day, and it confirms the rapid recovery from this type of minimally invasive surgery.

It is our opinion that excellent pain management in the early couple of postoperative days with the multi-modal analgesia approach encourages rapid recovery and reduction of morphine use during the hospital stay. It also reduces the need to be prescribed prolonged usage of opiates after discharge home from hospital. In order to see if there was any association between the use of intrapleural analgesia and the required supplemental opiate analgesia, it would be necessary to perform a prospective cohort or randomised trial comparing patient-controlled analgesia with and without intrapleural catheter usage. Surgeons may wish to expand and validate the use of the intrapleural analgesia technique for use with open or minimally invasive anterior scoliosis correction or anterior release procedures prior to posterior instrumented fusion surgery.

## Conclusions

This study has demonstrated a safe method of providing effective analgesia directly onto the spinal levels involved in thoracoscopic anterior scoliosis correction using a local anaesthetic bolus via an intrapleural catheter inserted in theatre. The results found that local anaesthetic boluses significantly decrease visual analogue pain scores and, when used in conjunction with multi-modal analgesia, resulted in ‘mild’ levels of pain in the immediate post-operative period following anterior scoliosis correction. The intermittent intrapleural analgesia method was not associated with any adverse events or complications and in part this can be attributed to the educated and well-coordinated care team consisting of nursing staff, physiotherapists, anaesthetic personnel and the surgical team.

## Competing interests

All authors declare they have no competing interests.

## Authors’ contributions

All the authors participated in the conception and design of the study. GNA and RDL performed the surgical procedures and were involved in critically reviewing the manuscript. SAC and MTI reviewed the literature, collected and collated the data and with CJA performed the statistical analysis, interpretation of the data and critically reviewing the manuscript. SAC and MTI drafted and revised the manuscript, with all authors critically reviewing the work regularly. All authors read and approved the final manuscript.
